# A Newly Documented Rare Case of Pachyonychia Congenita II in a Three-Month-Old Baby

**DOI:** 10.1155/crdm/8876939

**Published:** 2025-07-12

**Authors:** Zeinab Youness, Marwa Hallal, Rita Makhoul

**Affiliations:** ^1^Department of Dermatology, Military Hospital, Beirut, Lebanon; ^2^Dermatology Department, Gilbert and Rose-Marie Chagoury School of Medicine, Lebanese American University, Byblos, Lebanon; ^3^Dermatology Department, Universite Catholique de Louvain, Namur, Belgium

**Keywords:** congenital teeth, keratin genes, *KRT17* gene, milia, onychodystrophy, pachyonychia congenita, palmoplantar hyperhidrosis

## Abstract

We report the case of a three-month-old boy presenting with dystrophic nails, hyperhidrosis, congenital natal teeth, and milia-like lesions on the nose, without a family history of pachyonychia congenita (PC). Genetic testing confirmed a heterozygous pathogenic mutation (c.275A > G) in the *KRT17* gene, establishing the diagnosis of PC Type II. PC is a rare genetic disorder affecting keratinization, with variable clinical manifestations that can complicate early recognition. This case highlights the importance of molecular testing and dermatologic expertise in diagnosing and managing PC.

## 1. Introduction

Pachyonychia congenita (PC) is a rare hereditary condition affecting keratinization, which results from an underlying genetic mutation in one of the five keratin genes: *KRT6A*, *KRT6B*, *KRT6C*, *KRT16*, or *KRT17* [1]. The most frequent mode of inheritance for this condition is autosomal dominant inheritance, with around 30% of cases attributed to de novo pathogenic variants. In addition, a few instances of autosomal recessive inheritance have also been reported [2]. PC was formerly divided into two subtypes: PC-1, also known as Jadassohn–Lewandowsky syndrome, which is associated with variants in *KRT6A* and *KRT16*, and PC-2, also known as Jackson–Lawler syndrome, which was linked to variants in *KRT6B* and *KRT17*. Nevertheless, due to observed similarities in molecular and clinical data between these subtypes, a molecular analysis–based classification was adopted [3, 4]. Here, we report the case of a three-month-old infant boy who presented with congenital natal teeth and onychodystrophy without a family history of PC. A diagnosis of PC Type II was confirmed via molecular genetic testing.

## 2. Case Report

A three-month-old newborn boy, born to nonconsanguineous parents, exhibited natal teeth and alterations in the nails. The parents also noted palmoplantar hyperhidrosis. Apart from physiological jaundice during the neonatal phase, which resolved without notable complications, the family's medical history was unremarkable.

**Figure 1:**
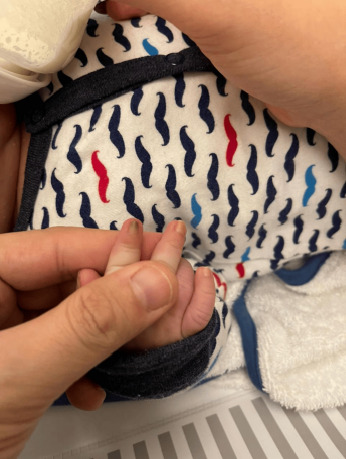
Onychodystrophy of the fingernails.

**Figure 2:**
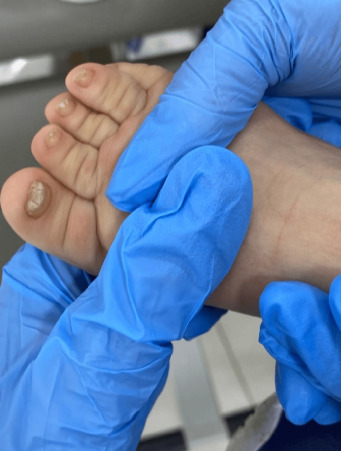
Onychodystrophy of the toenails.

**Figure 3:**
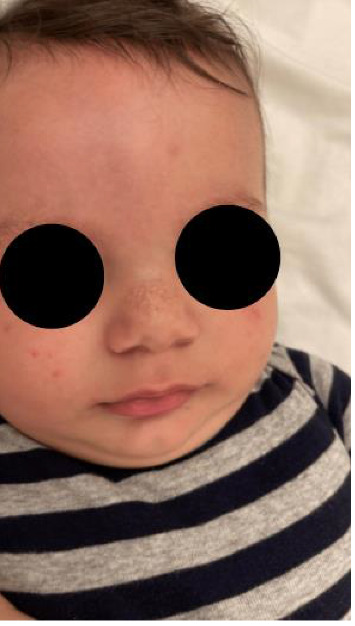
Milia on the nose.

**Figure 4:**
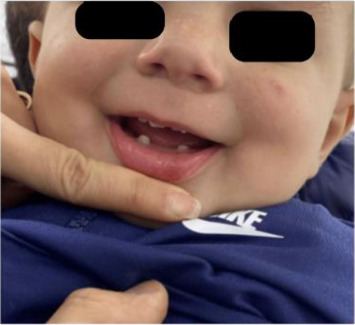
Lower incisors present since birth.

A physical examination revealed alterations in the nails of the first, second, and third fingers of the right hand; the first and second fingers of the left hand; as well as in the toes bilaterally (Figures 1 and 2). The nail plates appeared thickened, with yellow to yellow–green discoloration; onycholysis, subungual hyperkeratosis, and narrowing at the distal end of the nail plate were also present. These findings were consistent with onychodystrophy. The palms and soles were normal, with no clinically evident keratoderma. In addition, epidermal inclusion cysts resembling milia were observed on the face, predominantly affecting the nose (Figure 3). Oral examination revealed two lower incisor teeth, which had been present since birth (Figures 3 and 4). The remainder of the physical examination was normal, without any notable abnormalities.

The differential diagnosis included twenty nail dystrophy, PC, and onychomycosis. The potassium hydroxide (KOH) smear and nail clipping culture results were negative, indicating the absence of a fungal infection, thus ruling out candidal or dermatophytic onychomycosis. Whole exome sequencing (WES) analysis was performed as part of the genetic study, revealing a heterozygous missense mutation (c.275A > G) in the *KRT17* gene. This finding was confirmed by Sanger sequencing, which demonstrated a de novo occurrence, as both parents tested negative for the variant. The identification of this mutation conclusively established the diagnosis of PC Type II, also known as Jackson–Lawler disease.

## 3. Discussion

PC is a rare genodermatosis characterized by defective keratinization that affects the hair, nails, skin, and mucosa. It is usually inherited as an autosomal dominant trait, but a de novo pathogenic variant is found in approximately 30% of the cases [4]. There are few documented cases of recessive inheritance in the literature, and to date, no genetically confirmed cases of PC with a recessive pattern have been reported [1, 2]. The majority of patients exhibit thickened dystrophic nails and experience painful plantar keratoderma. Onychodystrophy is the most consistent physical finding observed in PC patients, with the nails usually being affected at birth or shortly after [4]. The thickening of the nail bed can lead to the development of “pincer” or “omega” deformity, as was observed in this case. Patients with *KRT6A* mutations tend to be more affected than those with other mutations. Plantar keratoderma typically begins when the child starts walking, with the formation of hyperkeratosis, callosities, and fissures, which contribute to plantar pain. Approximately 50% of the cases report hyperhidrosis or excessive sweating, which may be present from birth, as observed in this case. It may also lead to the formation of subepidermal blisters [4]. In addition, oral leukokeratosis is present in approximately 70% of the cases, with nearly half of these instances showing onset from birth [3]. In infants, this condition may be misdiagnosed as *Candida albicans*, leading to feeding difficulties [4]. Notably, individuals carrying *KRT6A* and *KRT17* genes experience a significantly earlier and heightened onset compared to those carrying *KRT6B* and *KRT16* genes [3]. Pilosebaceous cysts and steatocysts have been documented in association with both types of PC, affecting around 41% of patients. These cysts are more frequently observed in *KRT17* gene mutation carriers and tend to emerge around puberty. Milia is present mostly on the face and nose of newborns, as observed in our case [3]. Furthermore, follicular keratosis manifests on the trunk and areas that are prone to friction, such as the elbows and knees. Its prevalence is higher in late childhood and adolescence but tends to diminish in severity with age, specifically during adulthood [4]. The occurrence of teeth erupting at birth, which is known as “natal teeth,” has been documented in individuals with PC, particularly in those carrying *KRT17* mutations, as was seen in our case. Interestingly, there are no reported instances of natal teeth in patients with *KRT16* or *KRT6B* mutations [3].

A diagnosis of PC can be established in an affected individual either by observing the clinical triad of dystrophic nails, plantar keratoderma, and plantar pain, or by identifying a heterozygous pathogenic mutation within one of the five keratin genes associated with PC: *KRT6A*, *KRT6B*, *KRT6C*, *KRT16*, and *KRT17* [4]. These characteristic signs typically manifest within the first year of life. However, due to misdiagnosis, 25% of cases are correctly identified [5]. The severity of PC findings varies both between families and within the same family carrying the same pathogenic variant, emphasizing the need for individualized care. A thorough assessment of PC entails a comprehensive clinical examination and genetic counseling. The clinical examination may reveal variations and indications of the PC subtype. For instance, the presence of natal or prenatal teeth strongly suggests a PC-2 phenotype, likely associated with a K6b/K17 mutation, while nail dystrophy at birth, particularly affecting all of the nails, is indicative of a PC-K6a or PC-K17 mutation. Conversely, the emergence of palmar keratoderma during childhood, especially when accompanied by later-onset features, may indicate PC-K16. Nevertheless, molecular genetic testing remains essential to confirm the diagnosis and further classify the disease. The c.275A > G (p.Asn92Ser) variant in *KRT17* is a missense mutation classified as pathogenic according to ACMG/AMP guidelines [6]. It is absent from the general population in gnomAD (allele frequency 0.0%), and no homozygotes have been reported. In silico analysis supports a deleterious effect, with a CADD score of 26.2. This variant has been previously associated with both PC and steatocystoma multiplex, further supporting its clinical significance. Re-examining the patient after genetic testing may be necessary to fully characterize the phenotype [5]. Genetic counseling is recommended, given that PC predominantly follows an autosomal dominant inheritance pattern with incomplete penetrance. Genetic counselors can provide carriers with insights into the autosomal dominant nature of the gene and its implications for family planning. It is worth noting that approximately 30% of PC cases originate from de novo pathogenic variants [4]. When parents are clinically unaffected, the likelihood of their future offspring inheriting the condition is minimal, though slightly higher than the general population due to the potential presence of germline mosaicism [4].

## 4. Conclusion

Abnormalities in keratin-related structures such as hair, nails, and mucosa are hallmarks of the rare genetic disorder PC. This case report highlights a distinct presentation of PC, deviating from the typical familial pattern, suggesting a de novo genetic mutation. The absence of a notable family history underscores the necessity of a thorough dermatological examination and genetic testing for an accurate diagnosis. Therefore, dermatologists should perform a comprehensive physical examination of both parents, although genetic testing may not be necessary in the absence of clinical signs. Once a diagnosis is confirmed, regular monitoring is essential to manage symptoms and improve the patient's quality of life.

## Data Availability

The data that support the findings of this study are available on request from the corresponding author. The data are not publicly available due to privacy or ethical restrictions.
